# Effect of Size and Heterogeneity of Samples on Biomarker Discovery: Synthetic and Real Data Assessment

**DOI:** 10.1371/journal.pone.0032200

**Published:** 2012-03-05

**Authors:** Barbara Di Camillo, Tiziana Sanavia, Matteo Martini, Giuseppe Jurman, Francesco Sambo, Annalisa Barla, Margherita Squillario, Cesare Furlanello, Gianna Toffolo, Claudio Cobelli

**Affiliations:** 1 Information Engineering Department, University of Padova, Padova, Italy; 2 Fondazione Bruno Kessler, Povo, Trento, Italy; 3 Department of Computer and Information Science, University of Genova, Genova, Italy; University of Otago, New Zealand

## Abstract

**Motivation:**

The identification of robust lists of molecular biomarkers related to a disease is a fundamental step for early diagnosis and treatment. However, methodologies for the discovery of biomarkers using microarray data often provide results with limited overlap. These differences are imputable to 1) dataset size (few subjects with respect to the number of features); 2) heterogeneity of the disease; 3) heterogeneity of experimental protocols and computational pipelines employed in the analysis. In this paper, we focus on the first two issues and assess, both on simulated (through an in silico regulation network model) and real clinical datasets, the consistency of candidate biomarkers provided by a number of different methods.

**Methods:**

We extensively simulated the effect of heterogeneity characteristic of complex diseases on different sets of microarray data. Heterogeneity was reproduced by simulating both intrinsic variability of the population and the alteration of regulatory mechanisms. Population variability was simulated by modeling evolution of a pool of subjects; then, a subset of them underwent alterations in regulatory mechanisms so as to mimic the disease state.

**Results:**

The simulated data allowed us to outline advantages and drawbacks of different methods across multiple studies and varying number of samples and to evaluate precision of feature selection on a benchmark with known biomarkers. Although comparable classification accuracy was reached by different methods, the use of external cross-validation loops is helpful in finding features with a higher degree of precision and stability. Application to real data confirmed these results.

## Introduction

In the last decade, transcriptome analysis performed with high-throughput microarrays has experienced a huge diffusion and has profoundly changed the approach to the study of complex diseases. In an experimental design, the data typically come from different subjects and phenotypes. The analysis of these data has proven extremely useful for the identification of biomarker genes and for the development of new physiologic hypotheses useful for answering diagnostic, prognostic and functional questions. However, for complex diseases such as cancer, the high-throughput analysis carried out in different laboratories and research centers has given different results, with limited overlap or reduced statistical significance [1], [2]. These differences are matters of important scientific discussions and, besides the different or poorly reproducible experimental protocols and analysis pipelines [3]–[5], are imputed to two main reasons:

1. Datasets often include small numbers of subjects (some tens) with respect to the number of variables (tens of thousands of genomic probes in human) [6], [7];
2. The most complex pathologies, such as cancer, are heterogeneous and multifactorial, as a result of the alteration of multiple regulatory pathways and of the interplay between different genes and the environment, rather than referable to a single dysfunctional gene like in monogenic diseases [8], [9]. A consequence of this is that data are characterized by many correlated features; different features may thus be selected under different settings.

Widely used methodologies for the identification of biomarkers using microarray data are based on computing differential gene expression by univariate statistical tests. Such approaches provide information on the effects of specific genes as individual features, whereas it is now widely recognized that the interplay between weakly up/down regulated genes, although not significantly differentially expressed, might be extremely important to characterize a disease status [10]–[11]. Machine learning algorithms are, in principle, able to identify multivariate nonlinear combinations of features and have thus the possibility to select a more complete set of experimentally relevant gene features. In this context, classification methods are often used to select biomarker genes from microarray data. In a recent study [12], classification performance of different methods was compared across different microarray studies in terms of ability to select biomarkers discriminating between two conditions. Besides reaching good classification accuracy, obtaining stable list of biomarkers is critical both to understand the results from a biological point of view and to gain sufficient reliability on potential targets of clinical and pharmaceutical applications. The stability issue in feature selection has received much attention recently [13]–[16]. In a recent contribution, He and Yu [17] review existing stable feature selection methods for biomarker discovery.

As shown in [18], biomarker stability and accuracy are associated to task difficulty, and higher stability is found for higher accuracy. However, it is in principle possible to have a lack of stability due to the presence of many highly correlated features, even with accuracy equal to one. A first contribution of this work is the comparison of different classification methods in terms of consistency of lists of candidate biomarkers and classification accuracy. To this purpose, three real microarray datasets monitoring breast cancer patients with positive and negative estrogen receptor status are used; we compare biomarker lists from the three datasets as well as sets of sub-lists of different sample size obtained from each dataset.

A slightly different issue, although related with list stability, is the precision of biomarker identification, i.e. the ability to select true biomarkers, defined as features biologically related to the physiological or clinical condition under study as cause or effect of it. A second contribution of our work is the generation of a simulated dataset to assess alternative methods' performance across multiple studies and varying number of samples, and to evaluate precision of feature selection on a benchmark with known biomarkers. We extensively simulate the effect of heterogeneity and variability on different sets of synthetic microarray data consisting of two balanced groups of 50, 20, 15 or 10 subjects. Sample heterogeneity characteristic of complex diseases is reproduced within the same group by simulating both intrinsic variability of the population and the alteration of regulatory mechanisms induced by the disease. Population variability is simulated by modeling evolution of a pool of subjects in terms of pairing, mutation and selection in order to generate individuals characterized by different genotypes. Then, a subset of this population undergoes alterations in regulatory mechanisms so to mimic the disease state; these perturbations are slightly different across the patients in the diseased group, so to reflect the lack of homogeneity among patients that is typically reported in the literature for complex diseases [8].

Different methods for binary classification and feature weighting and ranking are applied to both simulated and real data. In particular, the classical Support Vector Machine algorithm (SVM) [19] is used in its linear and Gaussian kernel versions, and the SVM weights are used for feature ranking. As an alternative, I-Relief [20] is also used as the feature ranking algorithm coupled with linear SVM. One method of totally different nature is also applied: the Spectral Regression version of the Discriminant Analysis algorithm (SRDA) both as a classifier and a feature weighting algorithm [21]. All methods make use of the Entropy-based version of the classical Recursive Feature Elimination procedure as ranking schema [22], [23]. In all experiments, external cross-validation loops with separate training and test phases are employed to avoid overfitting effects such as selection bias [24]. Results are also compared with those obtained by using SAM, a widely applied variant of univariate statistical t-test [25].

## Methods

### Simulation of population variability

Each subject in the dataset was modeled by a regulatory network of *N* = 10000 genes, based on the simulator described in [26], using default parameter settings. The topology is characterized by the connectivity matrix *W*, with weights *w_ij_* different from zero if gene-product *j* directly affects the expression of gene *i*. The sign and the magnitude of *w_ij_* indicate the sign and the strength of the regulation. Differential equations were used to model the dynamics of transcription and degradation as continuous variables and to describe transcription delay with different time constants for each gene (see Text S1 for further details).

In molecular biology, transcription factors and enhancers are proteins that bind to specific DNA sequences and can regulate transcription of a gene by respectively activating/blocking the transcription and tuning the quantity of RNA transcribed in a unit of time. Loosely speaking, weights *w_ij_* of the connectivity matrix *W* can be interpreted as the affinity of the genome specific sequences for a transcription factor or an enhancer *j*, regulating expression of a gene *i*. Since weights *w_ij_* can in principle be mapped to specific nucleotide sequences in the genome, *W* can be interpreted as part of the genotype of the subject. Moreover, since each network is characterized by a finite number of attractors, reachable from a specific set of initial conditions and/or external stimuli, each attractor can be interpreted as the phenotype of an individual in a particular environmental condition.

Following these concepts, evolution of a population of *M* = 1000 individuals was simulated using a procedure similar to the one described in [27]. In summary, subjects were modeled as regulatory networks of *N* = 10000 nodes characterized by a specific genotype (the connectivity matrix *W* with weights *w_ij_*) and a specific phenotype (the system attractors). Given specific initial conditions (i.e. environment condition that we consider fixed for the purpose of this work), the initial population at generation 1 consisted of *M* individuals with identical connectivity matrix *W* and with *N* dimensional vectors of expression values obtained by considering the steady state reached by the system. Gene specific kinetic parameters *α_i_* and *β_i_* were sampled from Gaussian distributions with means *μ_α_*, *μ_β_* and standard deviations *σ_α_*, *σ_β_*. For each subject, *μ_α_* and *μ_β_* were set to 20 and 0.2, respectively, whereas *σ_α_* and *σ_β_* were sampled from a Gaussian distribution with means 0.5 and 0.02 and standard deviations equal to 0.075 and 0.0025, respectively. Parameters values (Text S1, Equations 1 and 2) were empirically chosen so to generate in silico data with statistical distribution similar to those observed on the real datasets.

To introduce genotype variability in the population, subsequent generations were produced by iteration of three steps: random *pairing* of individuals, *mutation* of a randomly chosen subset of subjects and *selection* of the surviving subjects. For computational reasons, these three steps were applied only to a sub-network of size *N* = 900, indicated as *W*_900_ in the following, which was constrained to be not connected to any of the other 9100 nodes in the network. Each step is described in detail in what follows.

*a. Pairing.* Offspring was created by randomly selecting two parents among the current population of *M* individuals and randomly combining rows of the connectivity matrix *W*_900_ from each parent with equal probability.

*b. Mutation.* Mutation was simulated by changing each nonzero *w_ij_* (which, by simulation, resulted equal to 1619 elements on a matrix of 900×900 = 810000 elements) with probability 0.025/1619. The new value of each mutated *w_ij_* was sampled from a Gaussian distribution with mean and standard deviation equal to 0 and 1, respectively. Therefore, at each iteration, each subject mutated with probability 0.025.

*c. Selection.* Assuming, in a naïve simplification of reality, that individuals behaved as haploid organisms and that the initial phenotype was essential for survival, subjects with at least one mutated *w_ij_* were allowed to survive only if their phenotype did not change with respect to the original population. In practice, we calculated the Euclidean distance between the expression profile of each mutated subject (the *N* dimensional vector of gene expression values at steady state) and the average expression profile of subjects at generation 1; if Euclidean distance exceeded the value of 0.81 (corresponding to the percentile 99.5 of the observed distances) the subject was eliminated, otherwise he/she survived. At each generation, *M* individuals were generated, independently of the number of parents survived in the previous generation. Evolution proceeded for a time sufficient to have a final population of *M* subjects with the same phenotype but different genotype, i.e. 150 generations (Figure S1).

Noise was added to expression data of the 10000 genes in the 1000 subjects as additive Gaussian noise with mean 0 and standard deviation sampled from the distribution of within-groups error variance in real datasets (paragraph 2.3), as described in [28]. In particular, the error variance associated to genes was approximated by a lognormal distribution with mean 0.22 and standard deviation 0.35.

### Simulated data

**Figure 1:**
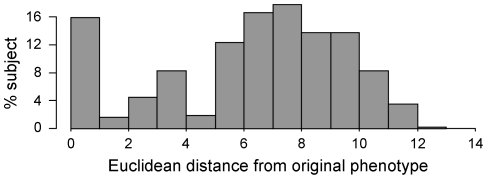
Variability of the diseased population. Histogram of the Euclidean distance between the steady states of the diseased population with respect to the original phenotype.

Once the base population was simulated, two groups, each of 500 subjects, were defined. The pathological condition was simulated by knocking out or down six target hubs, defined as those genes with the highest out-degree and expression value at steady state higher than 0.88, so that their knock-out (down) achieved an effect. The knock-out of gene *j* was simulated by setting to 0 its expression and all the elements of row *j* in matrix *W*. Consistently, the knock-down of gene *j* was simulated by halving its value and all the elements of row j in matrix *W*. Diseased subjects had 4, 5 or 6 genes belonging to *W*_900_ that were knocked out or down. The proportion of subjects with 4, 5 or 6 genes affected was set equal to 1/3, 1/3, 1/3, respectively. For each gene, the proportion of subjects affected by knock-out and knock-down was set equal to 1/3 and 2/3, respectively. Figure 1 displays the diseased group variability in terms of histogram of the Euclidean distance between the steady states of the original and the diseased population. The variability rises from both the intrinsic population variability, i.e. the different connectivity weights w_ij_ in *W*_900_, and the heterogeneity of the disease. Comparison between simulated and Affymetrix data (GSE2990, see below) showed that the datasets have very similar distribution (Wilcoxon test p-value equal to 0.9).

The putative biomarkers were defined as those genes directly or indirectly regulated by at least one of the six hubs, having expression modified by the knock-out (down). This resulted in 155 biomarkers on a total of 10000 features.

To consider the effect of sample size, we partitioned the two groups of 500 healthy and 500 diseased subjects into 4 sets of 10 balanced non-overlapping datasets of size 50, 20, 15 or 10 subjects per group (10 datasets for each case study), for a total of 40 simulated datasets.

### Real data

Publicly available data from three breast cancer microarray studies were collected from Gene Expression Omnibus repository (GEO) with accession numbers: GSE2990 [29], GSE3494 [30] and GSE7390 [31].

Datasets were all hybridized using Affymetrix U133 Genechips™ (HG-U133A). Samples that have known estrogen-receptor (ER) status were selected so to have balanced groups (ER+ and ER−), homogeneous with respect to characteristics such as age, tumor size and histological grade. We chose to investigate the ER status because it is always assessed in breast biopsies, therefore it is very often present among the clinical/pathological information given with the datasets. Moreover, the assessment of the ER status is important to divide breast cancer into molecular classes and to treat cancer with the hormone blocking therapy [32]. Since there are subgroups of samples belonging to multiple datasets, redundant subjects were removed. The resulting datasets are characterized by 22207 features (probe sets) and 66 subjects for GSE2990 (33 ER+, 33 ER−), 50 subjects for GSE3494 (25 ER+, 25 ER−) and 92 subjects for GSE7390 (46 ER+, 46 ER−). Comparison among the three datasets allowed assessing list stability in a real case study. To assess list stability within dataset, thus not accounting for experimental setup variability, and to compare the effect of sample size with simulated data, 20 subjects per ER status were repeatedly sampled from datasets GSE2990 and GSE7390 to set up smaller balanced datasets (10 datasets for each case study). Gene expression intensity signal was derived and normalized independently for each dataset using the robust multiarray average (RMA) algorithm [33]. Probe sets related to the estrogen receptor (ESR1) were removed from all datasets, since ESR1 is the gene more directly associated with ER status and can mask other potential descriptors of the underlying pathophysiology [34].

### Biomarker discovery methods

#### Support Vector Machine (SVM)

Support Vector Machines [19] are a set of supervised learning methods used for classification, in principle able to identify nonlinear features thus providing a more complete set of relevant genes. They were used here with linear (LSVM) and Gaussian kernel (GSVM). The tuning phase required the identification of the optimal value of the regularization parameter *c* (the trade-off between empirical error and smoothness of the solution) and, for the Gaussian kernel, of the bandwidth *σ*.

#### Iterative-Relief and SVM (IRSVM)

Iterative Relief [20] is a feature selection/ranking algorithm that solves a convex optimization problem with a margin-based objective function in a nearest-neighbor based strategy. The ranking provided by I-Relief can be used by an independent classifier: in our case, we used it together with linear SVM. The only required parameter to set is the bandwidth *σ* of the internal kernel.

#### Spectral Regression Discriminant Analysis (SRDA)

SRDA algorithm embeds the classical Discriminant Analysis into a regression framework through the use of spectral graph analysis [21]. This improves computational efficiency by solving only a set of regularized least squares problems without eigenvector computation involved. Moreover, the score attributed to each feature can be interpreted as a feature weight, allowing directly feature ranking. The regularization value α is the only parameter we had to tune.

#### Parameter Tuning

For GSVM, IRSVM and SRDA, parameter tuning was performed through a preliminary 3-fold cross-validation (without feature ranking) run for a set of possible parameter values.

#### Bootstrap

The four methods, LSVM, GSVM, IRSVM and SRDA, were used both in single cross-validation and in a Monte Carlo bootstrap resampling schema with B = 100 external training/test splits with 3-fold cross-validation as internal resampling (methods named as LSVM_B, GSVM_B, IRSVM_B and SRDA_B in the following). This strategy has been proved to be an effective countermeasure against unwanted selection bias effects [23], [24].

#### Ranking and selection

In the four aforementioned methods, the Entropy-based Recursive Feature Elimination (ERFE) procedure was used as the ranking schema [22]. Starting from the classical RFE algorithm [35], ERFE adaptively discards a subset of the least informative features according to an entropy measure of the distribution of the weights generated by the feature weighting schema. This guarantees a relevant speed-up of the ranking procedure without performance degradation. The optimal number of features was chosen in correspondence to the minimum classification error estimate.

#### Statistical analysis of Microarrays (SAM)

The SAM test [25] is a widely used univariate statistical test for the identification of differentially expressed genes from microarray data. This variant of the t-test accounts for the non Gaussian distribution of data. SAM uses a resampling procedure to derive the null hypothesis distribution and the false discovery rate (FDR) to account for multiple testing [36]. In this study, a FDR = 5% was used to select features after a ranking based on their p-value.

### Algorithm evaluation

Algorithm performance was evaluated in terms of the ability to select true biomarkers, to provide stable lists of biomarkers and to accurately classify the subjects.

The ability to select the true biomarkers was evaluated in term of precision (number of true positives divided by the number of selected features) obtained by the different methods according to their choice of the optimal number of features. The area under the precision *vs.* recall (number of true positives divided by the number of true biomarkers) curve was also considered to outline the ability of the different methods to rank the features, a task related with the ability to select the true biomarkers.

To evaluate the ability of the different methods to provide stable lists of biomarkers, the algebraic stability indicator derived by Canberra distance was used [15]. In particular, given two ordered lists *T1* and *T2* of *p* ranked features, the Canberra distance between them is defined as:
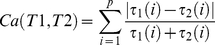
(1)where *τ_1_(i)* and *τ_2_(i)* indicate the rank, i.e. the position, of feature *i* in the ordered lists *T1* and *T2*, respectively. The stability indicator for a given set of lists was computed as the mean of the Canberra distances between pairs of lists in the set, normalized by its expected value on the whole permutation group on *p* features: the obtained value ranges then between 0 (maximal stability) and 1.4 (maximal instability), with 1 as the case of randomly generated lists. A different extension based on quotients of permutation groups allowed comparing lists *T1* and *T2* of different length *l_1_*, *l_2_*:
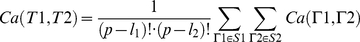
(2)where *p* is the total number of analyzed features and *Γj* (j = 1,2) belong to the set *Sj* of all the lists having the first *l_j_* features ordered as in *Tj* and the remaining (*p*–*l_j_*) elements ordered in all the (*p*–*l_j_*)! possible combinations. This is called the complete version of the partial lists distance: neglecting its component depending only on the discarded features we ended up with a different measure (called core distance) better tailored to highlight variations on partial short lists [37]. Full statements and proofs of the mathematical properties of the Canberra distance can be found in [38].

The Matthews correlation coefficient, *MCC* [39], was used as a measure of the quality of binary classifications. The MCC can be calculated directly from the confusion matrix using the formula:
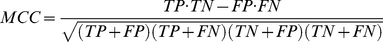
(3)In this equation, TP is the number of true positive, TN the number of true negative, FP the number of false positive and FN the number of false negative subjects.

Statistical significance of the comparison between each method and its bootstrap variant was assessed using Wilcoxon signed ranks test with significance level α equal to 0.05. Differences among the four multivariate feature selection methods in their bootstrap variant were assessed using Friedman test (α = 0.05), followed, if significant, by Wilcoxon signed ranks test to examine between which methods the differences actually occur, with a significance level α equal to 0.05/6 = 0.0083 to correct for multiple testing. Finally, SAM was compared with the other eight methods using Wilcoxon signed ranks test with a significance level α equal to 0.05/8 = 0.00625 to correct for multiple testing.

## Results

### Simulated data

Application of the nine biomarker discovery methods on the forty simulated datasets provides information on precision of feature selection, stability of biomarker lists and classification accuracy.

#### Feature selection

**Figure 2:**
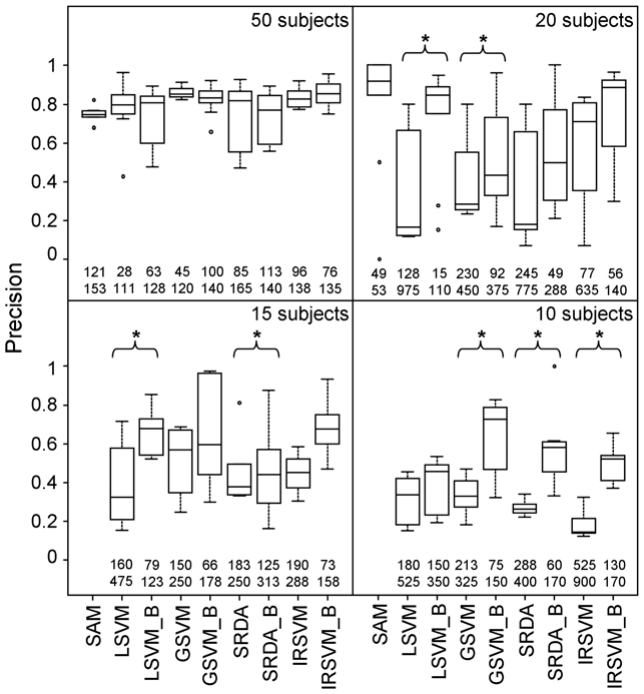
Precision of feature selection on simulated data. Boxplots of precision corresponding to the optimal number of features chosen by different methods when 50, 20, 15 or 10 subjects per group are available. A star highlights the significant differences between pair of bootstrap and non-bootstrap approaches (p-value lower than 0.05, Wilcoxon test). The interquartile range of the number of selected features is also reported below each boxplot.

Figure 2 shows boxplots of precision, obtained by the different methods according to their choice of the optimal number of features. Feature selection results show that bootstrap resampling schema leads to an improvement in terms of precision, statistically significant when the sample size decreases. In particular, with 20, 15 and 10 subjects per group, bootstrap improves precision of 1.5, 1.4 and 2 fold change, respectively (average improvement across the four different classification methods). Differences between bootstrap and non-bootstrap approach are statistically significant (p-value lower than 0.05, Wilcoxon signed ranks test) for LSVM and GSVM with 20 subjects per group, for LSVM and SRDA with 15 subjects per group, for all methods but LSVM with 10 subjects per group. In Figure 2, the interquartile range of the number of selected features is also reported. Interestingly, with less than 50 subjects per group, the bootstrap approaches have the tendency to select a lower number of features.

There are no appreciable differences among different bootstrap methods in terms of precision (Friedman test p-value always above 0.05 for every sample size). In the case of 50 subjects per group, SAM detects differentially expressed features with average precision comparable to that obtained by the other methods, but GSVM, IRSVM and IRSVM_B, which perform statistically significantly better than SAM (p-value equal to 0.002, 0.006, 0.006 respectively, Wilcoxon signed ranks test). With 20 subjects per group, SAM is not able to select any gene with FDR lower than 0.05 in six datasets, whereas in the remaining four, it selects in average 50 features with high precision (0.85 in average). In these latter cases, SAM performs statistically significantly better than LSVM (p-value = 0.004) and SRDA (p-value = 0.006), i.e. two methods without the bootstrap approach. Finally, with less than 20 subject per group, SAM is not able to select any gene in any of the dataset with FDR lower than 0.05; thus we could not report any result in these latter two cases.

**Figure 3:**
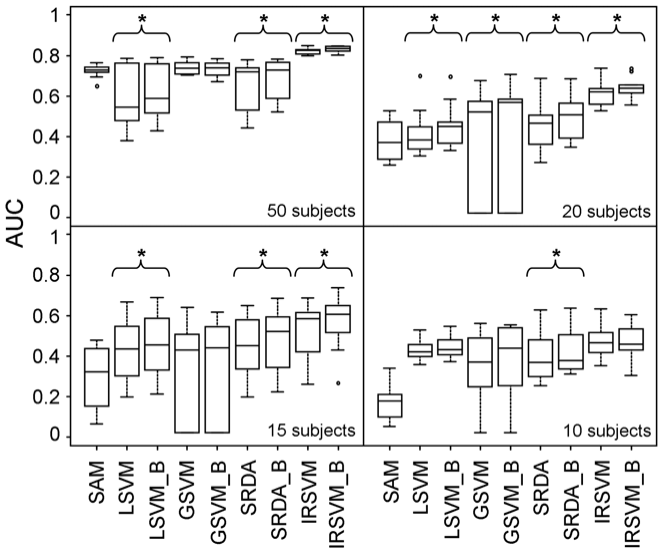
Evaluation of feature ranking on simulated data. Boxplots of area under the precision *vs.* recall curves obtained by ranking features according to the different methods, when 50, 20, 15 or 10 subjects per group are available.

A slightly different task, although related to feature selection, is feature ranking. In principle, a method could rank features properly, but fail to select the optimal number of features. Areas under the precision *vs.* recall curves (AUC) obtained by ranking features (Figure 3) show appreciable differences between methods. Bootstrap methods perform better than their standard variants for datasets of size 50, 20 and 15, for all methods (p-value always below 0.005) but GSVM. For datasets of size 10, only SRDA_B improves with respect to SRDA (p-value = 0.01). With datasets of 50, 20 and 15 subjects per class, IRSVM_B is the best performing algorithms (Friedman test gave p-value lower than 0.004 for every sample size and Wilcoxon signed ranks test gave p-value lower than 0.003 for every comparison between IRSVM_B and the other bootstrap methods). With 10 subjects per group, all multivariate methods show AUC below 0.5, without statistically significant differences among them.

With 50 and 20 subjects per group, a simple univariate test such as SAM is able to rank differentially expressed features with performance comparable to multivariate methods such as LSVM, GSVM, SRDA and their bootstrap versions, but not to IRSVM and IRSVM_B that perform better (p-values equal to 0.002 for both tests). However, when the number of subjects is lower than 20, SAM performance in feature ranking dramatically drops with respect to classification based methods (p-value lower than 0.002 for all comparisons but GSVM and GSVM_B). This behavior is consistent with the inability of SAM to select any feature with 15 and 10 subjects per group.

#### Feature stability

**Figure 4:**
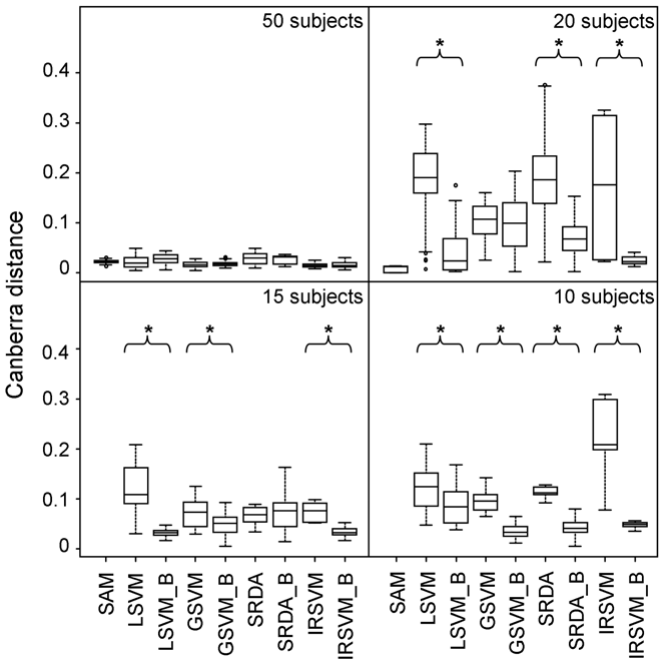
Evaluation of feature stability on simulated data. Boxplots of the core Canberra distance between lists of selected features obtained using different methods when 50, 20, 15 or 10 subjects per group are available. A star highlights the significant differences between pair of bootstrap and non-bootstrap approaches (p-value lower than 0.05, Wilcoxon test).

The ability of the various methods to select the same features across different datasets is depicted in Figure 4, where the boxplots of the core Canberra distance (Equation 2) of the lists of selected features are shown. The distance between the ranked lists increases for all the methods when the number of subjects per group decreases. Results are consistent with those obtained for feature selection: the bootstrap resampling schema leads to an improvement in list stability, statistically significant when sample size decreases. In particular, differences are statistically significant for LSVM, SRDA and IRSVM with 20 subjects per group (p-value always lower than 0.036), for LSVM, GSVM and IRSVM with 15 subjects per group (p-value always lower than 0.033), for all methods with 10 subjects per group (p-value always lower than 0.001). Among bootstrap approaches, IRSVM_B is the best performing method in terms of list stability, when 20 subjects per group are available; LSVM_B performs as IRSVM_B in the case of 15 subjects per group; GSVM_B performs as IRSVM_B in the case of 10 subjects per group (Friedman test gave p-value lower than 10^−11^ for sample size 20, 15, 10 and Wilcoxon signed ranks test gave p-value lower than 0.001 for every significant pairwise comparison).

In the case of 50 subjects per group, SAM shows list stability comparable to the one obtained by the other methods. With 20 subjects per group, SAM is as good as IRSVM_B; however, results are limited to the four datasets for which SAM was able to select features below the 0.05 FDR threshold. As for feature selection, with less than 20 subjects per group we do not report any results since SAM was not able to select any gene in any of the dataset.

#### Classification Accuracy

Bootstrap approach also improves classification accuracy (Table 1): with 50 subjects per group LSVM_B and IRSVM_B perform better than their standard versions (p-value equal to 0.019 and 0.007, respectively); with 20 subjects per group GSVM_B and SRDA_B perform better than their standard versions (p-value equal to 0.030 and 0.025, respectively); with 15 subjects per group LSVM_B, GSVM_B and SRDA_B perform better than their standard versions (p-value equal to 0.031, 0.031 and 0.016, respectively). All bootstrap classification methods perform equally well (Friedman test p-values always above 0.15 for every sample size) in terms of classification accuracy.. SAM was excluded from this part of the analysis.

**Table 1:** MCC corresponding to the optimal number of features obtained using different methods - simulated data.

	50	20	15	10
**LSVM**	0.73 (0.62, 0.82)	0.69 (0.51, 0.93)	0.73 (0.60, 0.88)	0.70 (0.60, 0.82)
**LSVM_B**	0.77 (0.65, 0.87)	0.74 (0.54, 0.95)	0.80 (0.68, 0.94)	0.73 (0.64, 0.83)
**GSVM**	0.78 (0.70, 0.87)	0.76 (0.62, 0.91)	0.81 (0.72, 0.89)	0.73 (0.66, 0.80)
**GSVM_B**	0.80 (0.65, 0.92)	0.81 (0.62, 0.95)	0.83 (0.66, 0.94)	0.71 (0.64, 0.86)
**SRDA**	0.75 (0.66, 0.84)	0.72 (0.61, 0.93)	0.74 (0.61, 0.87)	0.69 (0.60, 0.80)
**SRDA_B**	0.77 (0.67, 0.85)	0.74 (0.59, 0.96)	0.75 (0.60, 0.94)	0.73 (0.61, 0.83)
**IRSVM**	0.77 (0.66, 0.84)	0.83 (0.61, 0.94)	0.77 (0.67, 0.85)	0.65 (0.60, 0.80)
**IRSVM_B**	0.81 (0.67, 0.92)	0.72 (0.50, 0.95)	0.80 (0.64, 0.94)	0.69 (0.51, 0.86)

Average MCC obtained when 50, 20, 15 or 10 subjects per group are available. Range of values is indicated in parenthesis.

### Real Data

Application of the various methods on breast cancer data provides information on list stability and classification accuracy.

**Figure 5:**
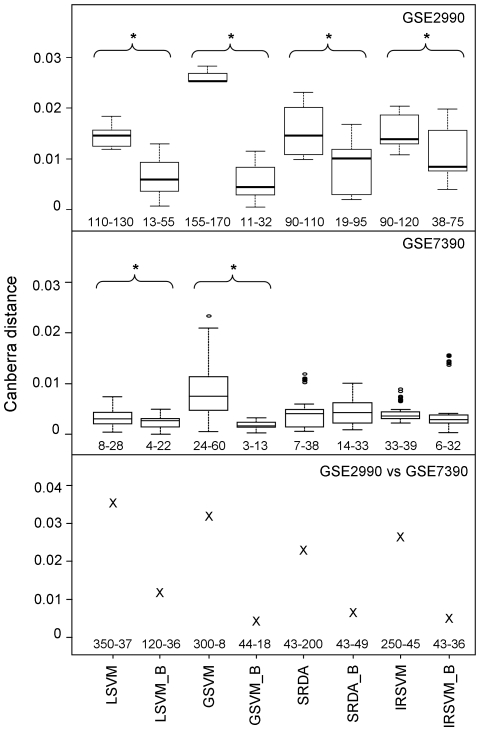
Evaluation of feature stability on real data. Boxplots of the core Canberra distance between lists of selected features provided by different classification methods when 20 subjects per group are repeatedly sampled from GSE2990 (upper panel) and GSE7390 (middle panel) datasets. A star highlights the significant differences between pair of bootstrap and non-bootstrap approaches (p-value lower than 0.05, Wilcoxon test). The interquartile range of the number of selected features is reported below each boxplot. The core Canberra distances between lists of biomarkers provided by different methods on the complete GSE2990 *vs.* GSE7390 datasets are shown in the lower panel together with the number of selected features in each dataset.

Results on dataset GSE3494 are not shown since none of the different methods gave good accuracy (MCC always below 0.4). On the other two datasets, results confirmed those obtained by simulated data. In particular, bootstrap resampling schema leads to an improvement in list stability (Figure 5), appreciable both when the complete datasets GSE2990 and GSE7390 are compared and when 20 subjects per group are repeatedly sampled from each dataset, for a total of 10 resampled dataset for each of the original datasets.

Differences between bootstrap and standard approach are statistically significant for every method (p-value always lower than 0.002) with dataset GSE2990 and for LSVM and GSVM with dataset GSE7390.

In terms of stability, SAM performance is poor: when 20 subjects per group are repeatedly sampled from each dataset the core Canberra distance between lists of biomarkers ranges between 0.04 and 0.37 (average 0.27) for GSE2990 and between 0.13 and 0.31 (average 0.22) for GSE7390; on the other hand, between the complete datasets (GSE2990 *vs*. GSE7390) the core Canberra distance is equal to 0.63. SAM results are not shown in Figure 5 to avoid masking the differences among the other methods.

The MCC obtained using different methods on real datasets is shown in Table 2. The first two columns report the MCC for GSE2990 and GSE7390, respectively, when 20 subjects per group are repeatedly sampled from each dataset. The third and fourth columns of Table 2 report the MCC obtained using the complete datasets GSE2990 and GSE7390. Results are comparable to those obtained using simulated data. Bootstrap approach improves classification accuracy on dataset 7390 for all methods (p-value equal to 0.02, 0.04, 0.001, 0.03 for LSVM_B, GSVM_B, SRDA_B and IRSVM_B, respectively, with respect to their standard version), whereas, with dataset 2990, the differences between bootstrap and standard approaches are not statistically significant. It is confirmed the tendency of the bootstrap approaches to select a lower number of features. As observed with simulated data, all bootstrap classification methods perform equally well in terms of classification accuracy (Friedman test p-values always above 0.06 on both the datasets).

**Table 2:** MCC corresponding to the optimal number of features obtained using different methods – real data.

	GSE2990 20 subjects	GSE7390 20 subjects	GSE2990	GSE7390
**LSVM**	0.64 (0.61, 0.69)	0.77 (0.61, 0.90)	0.60	0.79
**LSVM_B**	0.65 (0.51, 0.77)	0.81 (0.58, 0.91)	0.68	0.81
**GSVM**	0.62 (0.59, 0.64)	0.73 (0.60, 0.83)	0.59	0.74
**GSVM_B**	0.65 (0.60, 0.71)	0.78 (0.61, 0.91)	0.61	0.77
**SRDA**	0.63 (0.61, 0.66)	0.74 (0.62, 0.85)	0.50	0.78
**SRDA_B**	0.67 (0.61, 0.78)	0.83 (0.66, 0.90)	0.67	0.77
**IRSVM**	0.62 (0.47, 0.69)	0.80 (0.65, 0.91)	0.60	0.78
**IRSVM_B**	0.67 (0.58, 0.82)	0.82 (0.62, 0.91)	0.67	0.81

Average MCC obtained when 20 subjects per group are available, sampled from datasets GSE2990 and GSE7390 MCC (range of values is indicated in parenthesis), and obtained on the complete datasets GSE2990 and GSE7390.

To improve our confidence in the biological meaningfulness of the results obtained with real data, the functional annotation of the selected genes was considered. In particular, we considered: 1) the intersection of the lists obtained by the four bootstrap methods on datasets GSE2990 and GSE7390; 2) the intersection of the lists obtained by IRSVM_B on datasets GSE2990 and GSE7390. The two lists of genes and the results of enrichment analysis are available in Text S2.

## Discussion

The identification of an appropriate and robust biomarker signature of a disease is a fundamental step for early diagnosis and treatment. However, for complex diseases such as cancer, high throughput analysis carried out in different research centers may exhibit poor reproducibility, with limited overlap or reduced statistical significance. The results of the MAQC-II study address in a comprehensive analysis this issue on real datasets by comparing methods and procedures between data analysis teams [18]. Here we have further explored the effect of the intrinsic complexity of the task.

A first contribution of this work is the comparison of different classification methods applied on real microarray datasets, in terms of consistency of lists of candidate biomarkers and classification accuracy. A second contribution of our work is the generation of a simulated dataset to extensively assess average method performance on a large number of studies and experimental conditions, and to evaluate precision and feature ranking performance on a benchmark with known biomarkers. Heterogeneity of samples in each group is obtained by simulating both intrinsic variability of the population and heterogeneity of the disease. Despite its simplicity with respect to real systems, the simulator provides a versatile test bed to assess a wide spectrum of methodologies. The dataset is available upon request (barbara.dicamillo@dei.unipd.it).

Results on simulated data show that when some tens of subjects are available per group, performance of different methods are comparable. However, when available subjects are equal or lower than 20, bootstrap resampling schema leads to an improvement in the precision of the selected features and list stability. Bootstrap approach slightly improves also classification accuracy when 50, 20 or 15 subjects per group are available. Among the different methods here considered, IRSVM_B provides the best combination of feature ranking and biomarker stability; moreover, it reaches the best average performance also in terms of classification accuracy.

In the case of 50 subjects per group, a simple univariate test such as SAM shows performance comparable to that obtained by the other methods. With 20 subjects per group, SAM performance strongly depends on the dataset: on the simulated data, for example, SAM is not able to select any gene with FDR lower than 0.05 in six datasets, whereas in the remaining four, it selects in average 50 features with high precision (0.85 in average) and stability comparable to the one obtained using IRSVM_B, although this latter outperforms SAM in feature ranking. Finally, with less than 20 subjects per group, SAM performance dramatically drops with respect to classification based methods.

With real data, only list stability and classification accuracy can be assessed. In both cases, results of classification methods tightly resemble those obtained with simulated data.

In conclusion, our analysis confirms the MAQC-II indication that comparably good classification accuracy can be reached by different methods on the same task, provided that a valid Data Analysis Plan is adopted [18]. Furthermore, we found a systematic improvement due to bootstrap in selecting features with a high degree of precision and stability. Overall, the crucial factor affecting list stability seems to be that the classification task is under constrained. When additional information is present on the relationships between genes, this information could be used to improve the stability with respect to the features of the classifiers. The basic idea of this strategy would be to take into account the complex gene relationships, instead of considering genes as independent features. In future works, we plan to compare the use of different biological information from genomic databases in the learning process by integrating different prior knowledge like functional annotations, protein-protein interactions, and expression correlation among genes.

## Supporting Information

Figure S1 **Progression of population mutation with generations.** Total number of subjects mutated with respect to the original population with the progress of generations. Only survived subjects are represented for each generation.
(TIF)

Text S1 **In Silico model of regulatory networks.**
(DOC)

Text S2 **Selected genes.**
(DOC)
