# Supplementary figures and images for: Effect of Size and Heterogeneity of Samples on Biomarker Discovery: Synthetic and Real Data Assessment
Source: PLoS One. 2012 Mar 5;7(3):e32200. doi: 10.1371/journal.pone.0032200 (PMC3293892; doi:10.1371/journal.pone.0032200)

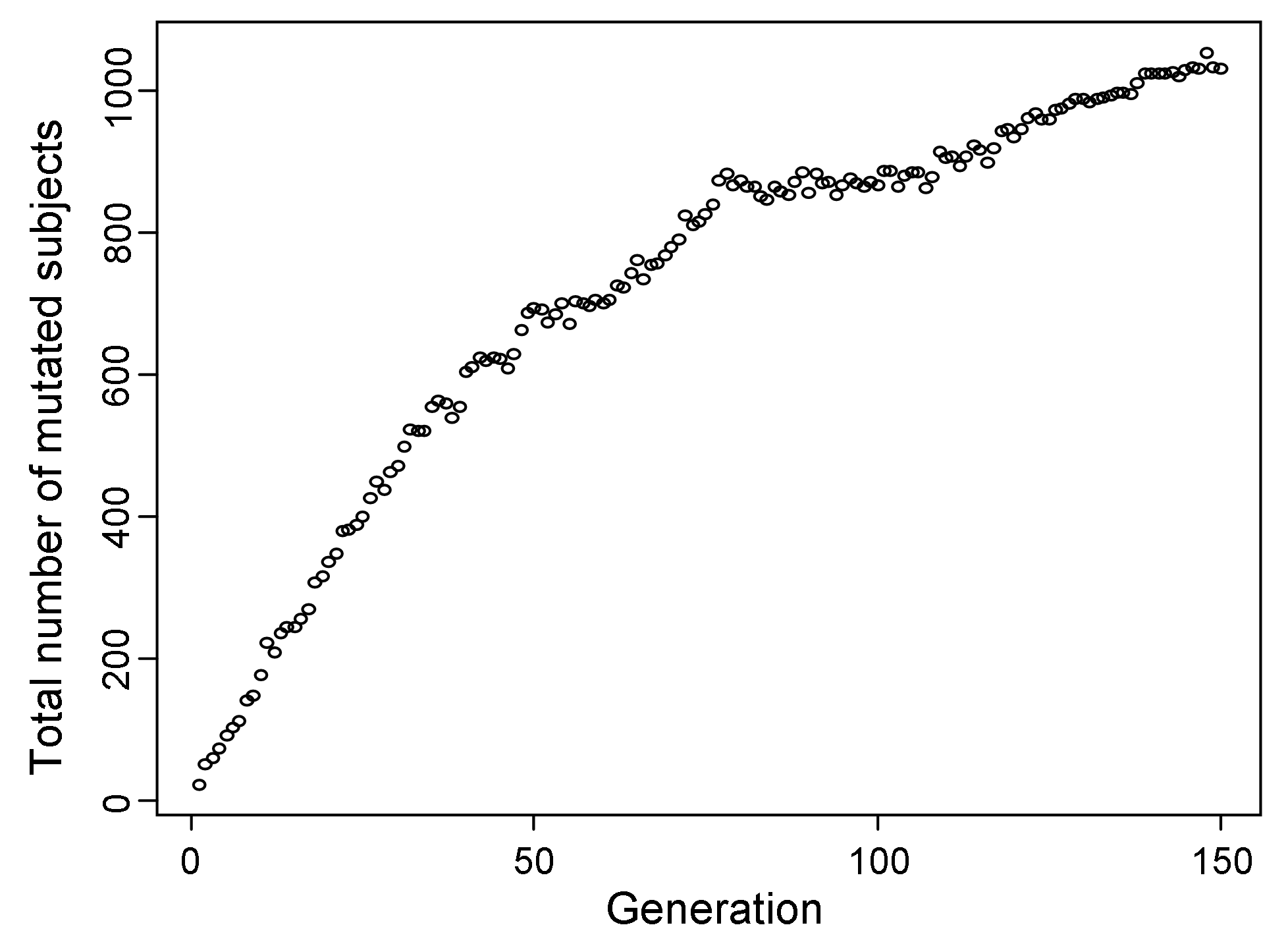

Supplement: Figure S1 — Progression of population mutation with generations. Total number of subjects mutated with respect to the original population with the progress of generations. Only survived subjects are represented for each generation. (TIF) [file pone.0032200.s001.tif]
