# Supplementary material for: Effect of Size and Heterogeneity of Samples on Biomarker Discovery: Synthetic and Real Data Assessment
Source: PLoS One. 2012 Mar 5;7(3):e32200. doi: 10.1371/journal.pone.0032200 (PMC3293892; doi:10.1371/journal.pone.0032200)
Supplement: Text S1 — In Silico model of regulatory networks. (DOC) [file pone.0032200.s002.doc]

in silico model of regulatory networks

Each subject in the dataset was modeled by a regulatory network of *N*=10000 genes, based on the simulator described in Di Camillo *et al*. (2009), using default parameter settings. Network topology was randomly generated with scale-free distribution of node degree and clustering coefficient independent of the number of nodes. The topology is characterized by the connectivity matrix *W*, with weights *w_ij_* different from zero if gene-product *j* directly affects the expression of gene *i*. The sign and the magnitude of *w_ij_* indicate the sign and the strength of the regulation. A target value *T_i_*(*W*,*t*) was derived for gene *i* at time *t* as a function of the different action of its regulators (*T_i_*(*W*,*t*) represents the expression value to which gene *i* tends at time *t* as an effect of the expression level of its regulators). By explicitly representing interactions among the regulators of each gene, the simulated systems were characterized by a finite number of basins of attractors, where each attractor corresponds to a steady state or a periodic behavior.

Differential equations were used to model the dynamics of transcription and degradation as continuous variables and to describe transcription delay with different time constants for each gene. In particular, the rate of change of the expression level of gene *i* at time *t* was described as:

$\frac{dx_{i}\left(t\right)}{dt}=\lambda_{i}\cdot\left[S_{i}\left(T_{i}\left(W,t\right),\alpha_{i},\beta_{i}\right)-x_{i}\left(t\right)\right]$ (1)

where *λ_i_* is a time constant influencing both the rate of transcription and the degradation term and *S_i_*(*T_i_*(*W*,*t*),*α_i_*,*β_i_*) is a sigmoid activation function, modulating the target value *T_i_*(*W*,*t*) and depending on gene *i* specific parameters *α_i_* and *β_i_*:

$S_{i}\left(T_{i}\left(W,t\right),\alpha_{i},\beta_{i}\right)=\frac{1}{1+\exp\left[-\alpha_{i}\cdot\left(T_{i}\left(W,t\right)-\beta_{i}\right)\right]}$ (2)

References

Di Camillo,B. *et al.* (2005) A quantization method based on threshold optimization for microarray short time series. *BMC Bioinformatics*, **6**(Suppl. 4), S11.
