# Supplementary material for: Effect of Size and Heterogeneity of Samples on Biomarker Discovery: Synthetic and Real Data Assessment
Source: PLoS One. 2012 Mar 5;7(3):e32200. doi: 10.1371/journal.pone.0032200 (PMC3293892; doi:10.1371/journal.pone.0032200)
Supplement: Text S2 — Selected genes. (DOC) [file pone.0032200.s003.doc]

SELECTED GENES

To improve our confidence in the biological meaningfulness of the results obtained with real data, the functional annotation of the selected genes was considered. In particular, we considered: 1) the intersection of the lists obtained by the four bootstrap methods on datasets GSE2990 and GSE7390 (list 1); 2) the intersection of the lists obtained by IRSVM_B on datasets GSE2990 and GSE7390 (list 2).

List 1 includes 35 probe sets corresponding to 28 genes (see Supplementary Table S1). Enrichment analysis performed using Gene Ontology (GO) terms (hypergeometric test, with significant level 0.05 not corrected for multiple testing) revealed that the selected genes are mainly involved in GO terms *response to stimuli* (chemical GO:0042221, endogenous GO:0009719 and stress GO:0006950), *developmental processes* (GO:0032502), *cell death and* *proliferation* (GO:0008283) and *migration* (GO:0016477)*.* Proteins associated to the selected genes are prevalently located in the nucleus, in the plasma membrane and in the cell projection. It is noteworthy that, within the *developmental process* (GO:0032502) and *multicellular organismal process* (GO:0032501) classes, several GO terms related to the nervous system are enriched, such as *nervous system development* (GO:0007399) and *transmission of nerve impulse* (GO:0019226)*.* This might provide some insights on the frequent occurrence of breast cancer metastases in the nervous system. The biological reliability of the results is confirmed by the fact that 17 out of 28 genes were already mentioned in relation with breast cancer. We here briefly comment on a subset of them that we consider particularly relevant. S100A8 is a calcium binding protein that is involved in the regulation of many cellular processes such as *cell cycle progression* and *differentiation* (Moon *et al.*, 2008). NFIB, in a fused form with MYB, was found as a hallmark of breast and other carcinomas (Persson *et al.*, 2009). IGFBP5 and IGF1R belong to the family of Insulin-like growth factors and they are both involved in the breast carcinogenesis process (Gu *et al.*, 2010, Grice *et al.*, 2010). TFF1 and TFF3 are both members of the trefoil family and they are known to be expressed in the gastro-intestinal mucosa, probably defending it from insults, stabilizing the mucus layer and affecting healing of the epithelium (Berthau *et al.*, 2007).

List 2 (features selected using IRSVM_B) contains 51 genes. Enrichment analysis revealed that they are mainly involved in GO terms regarding *compartment of the* *cell nucleus,* the *cytoplasm* (GO:0005737)*,* *plasma membrane* (GO:0005886) and *cell projection* (GO:0042995)*.* The most enriched GO terms within the biological process domain are: *response to stimuli* (chemical, GO:0042221, endogenous, GO:0009719), *nervous system development* (GO:0007399), *cell proliferation* (GO:0008283), those terms related to the regulation of cell death and those regulating the cellular motility. Interestingly, several GO terms belonging to the *regulation of biological quality* (GO:0065008) are enriched, such as the *regulation of anatomical structure size* (GO:009006) and those terms involved in the *homeostasis*. 27 out of 51 genes in the list were previously associated to breast cancer in several studies. A subset of genes of biological interest and not included in list 1 deserves further comments: S100A9 is a calcium binding protein paired with S100A8 (Moon *et al.*, 2008). MYB is a transcription factor involved with NFIB in several carcinomas including breast cancer (Persson *et al.*, 2009). GSTM3 is a gluthatione S-transferase protein that belongs to the mu-class enzymes. Genetic variations occurring in the mu-class have been associated to changes in the individual’s susceptibility to carcinogens and toxins (Yu *et al.*, 2010). ADM may function as hormone in circulation control, being highly concentrated in the blood. It is also know to be highly expressed in several tissues (Berthau *et al.*, 2007).

References

Berthau P. *et al.* (2007) Exquisite sensitivity of TP53 mutant and basal breast cancers to a dose-dense epirubicin-cyclophospamide regimen. *PLoS Med.* 4(3):e90.

Moon A. *et al.* (2008) Global gene expression profiling unveils S100A8/A9 as candidate markers in H-ras-mediated human breast epithelial cell invasion. *Mol. Cancer. Res. USA*, **10**, 1544-53.

Persson M. *et al.* (2009) Recurrent fusion of MYB and NFIB transcription factor genes in carcinomas of the breasts and head and neck. *Proc. Nat. Acad. Sci.* *USA*, **106**(44) 18740-4.

Grice D.M. *et al.* (2010) Golgi calcium pump secretory pathway calcium ATPase1 (SPCA1) is a key regulator of insulin-like growth factor receptor (IGF1R) processing in the basal-like breast cancer cell line MDA-MB-231. *J Biol. Chem.* 285(48) 37458-66.

Gu F. *et al.* (2010) Eighteen insulin-like growth factor pathway genes, circulating levels of IFG-I and its binding protein, and risk of prostate and breast cancer. *Cancer Epidemiol. Biomarkers Prev.* 19(11) 2877-87.

Yu K.D. *et al.* (2010) Genetic variants in GSTM3 gene within GSTM4-GSTM2-GSTM1-GSTM5-GSTM3 cluster influence breast cancer susceptibility depending on GSTM1. *Breast Cancer Res. Treat.* 121(2) 485-96.

**Table S2.1**Genes in the list 1 and the corresponding enriched GO terms. Genes contained also in list 2 are marked in bold fonts.

| Gene Name | # terms | GO terms |
| --- | --- | --- |
| **TFF1** | 14 | GO:0009725, GO:0009719, GO:0010039, GO:0010033, GO:0048545, GO:0043434, GO:0043627, GO:0042221, GO:0001894, GO:0060249, GO:0048871, GO:0065008, GO:0010038, GO:0005488 |
| **IGF1R** | 30 | GO:0009725, GO:0009719, GO:0010033, GO:0006916, GO:0043434, GO:0030334, GO:0030335, GO:0040012, GO:0051272, GO:0040017, GO:0051270, GO:0042221, GO:0043069, GO:0043066, GO:0007399, GO:0060548, GO:0031175, GO:0008283, GO:0016477, GO:0048812, GO:0048732, GO:0048523, GO:0019838, GO:0042802, GO:0019899, GO:0005488, GO:0042995, GO:0043005, GO:0044459, GO:0005886 |
| **TFF3** | 6 | GO:0009725, GO:0009719, GO:0010033, GO:0043434, GO:0042221, GO:0005737 |
| ADCY9 | 8 | GO:0009725, GO:0009719, GO:0010033, GO:0042221, GO:0016829, GO:0005488, GO:0044459, GO:0005886 |
| **GATA3** | 7 | GO:0009725, GO:0009719, GO:0010033, GO:0048545, GO:0043627, GO:0042221, GO:0005488 |
| GSTM3 | 10 | GO:0009725, GO:0009719, GO:0010033, GO:0048545, GO:0043627, GO:0042221, GO:0007399, GO:0042802, GO:0005488, GO:0005737 |
| BCL2 | 47 | GO:0009725, GO:0009719, GO:0014910, GO:0010039, GO:0010033, GO:0014909, GO:0048545, GO:0014812, GO:0006916, GO:0043434, GO:0030334, GO:0030335, GO:0043627, GO:0040012, GO:0051272, GO:0040017, GO:0051270, GO:0042221, GO:0043069, GO:0043066, GO:0007399, GO:0060548, GO:0001894, GO:0031175, GO:0060249, GO:0048871, GO:0042493, GO:0051240, GO:0008283, GO:0065008, GO:0016477, GO:0048589, GO:0048812, GO:0007015, GO:0090066, GO:0048732, GO:0048523, GO:0010038, GO:0042802, GO:0019904, GO:0019899, GO:0005488, GO:0044459, GO:0005886, GO:0005829, GO:0044428, GO:0005737 |
| **ERBB4** | 32 | GO:0009725, GO:0009719, GO:0010033, GO:0048545, GO:0006916, GO:0045768, GO:0030334, GO:0030335, GO:0040012, GO:0051272, GO:0040017, GO:0051270, GO:0045767, GO:0042221, GO:0043069, GO:0043066, GO:0007399, GO:0060548, GO:0001894, GO:0060249, GO:0048871, GO:0042493, GO:0051240, GO:0008283, GO:0065008, GO:0016477, GO:0048732, GO:0048523, GO:0005488, GO:0044459, GO:0005886, GO:0005737 |
| ADM | 21 | GO:0009725, GO:0009719, GO:0010033, GO:0048545, GO:0006916, GO:0043434, GO:0045768, GO:0045767, GO:0042221, GO:0043069, GO:0043066, GO:0060548, GO:0031175, GO:0051240, GO:0008283, GO:0065008, GO:0048812, GO:0090066, GO:0048523, GO:0005488, GO:0005737 |
| IL6ST | 27 | GO:0014910, GO:0010033, GO:0014909, GO:0014812, GO:0006916, GO:0045768, GO:0030334, GO:0030335, GO:0040012, GO:0051272, GO:0040017, GO:0051270, GO:0045767, GO:0042221, GO:0043069, GO:0043066, GO:0060548, GO:0051240, GO:0008283, GO:0065008, GO:0016477, GO:0048523, GO:0019838, GO:0042802, GO:0005488, GO:0044459, GO:0005886 |
| **IGFBP5** | 14 | GO:0014910, GO:0014909, GO:0014812, GO:0030334, GO:0040012, GO:0051270, GO:0008283, GO:0065008, GO:0016477, GO:0048589, GO:0090066, GO:0048523, GO:0019838, GO:0005488 |
| **ABAT** | 13 | GO:0010039, GO:0010033, GO:0042221, GO:0042493, GO:0065008, GO:0010038, GO:0042802, GO:0019899, GO:0005488, GO:0070013, GO:0043233, GO:0031974, GO:0005737 |
| **SON** | 12 | GO:0006916, GO:0043069, GO:0043066, GO:0060548, GO:0048523, GO:0019904, GO:0005488, GO:0031981, GO:0070013, GO:0044428, GO:0043233, GO:0031974 |
| PDE4A | 10 | GO:0042221, GO:0042493, GO:0005488, GO:0042995, GO:0031252, GO:0044459, GO:0005886, GO:0044463, GO:0005829, GO:0005737 |
| **FABP7** | 5 | GO:0007399, GO:0008283, GO:0048523, GO:0005488, GO:0005737 |
| EVL | 10 | GO:0007399, GO:0007015, GO:0019904, GO:0005488, GO:0042995, GO:0031252, GO:0044459, GO:0005886, GO:0005829, GO:0005737 |
| **MYO6** | 17 | GO:0007399, GO:0031175, GO:0065008, GO:0005488, GO:0042995, GO:0031252, GO:0043005, GO:0044459, GO:0031981, GO:0005886, GO:0044463, GO:0070013, GO:0005829, GO:0044428, GO:0043233, GO:0031974, GO:0005737 |
| **GFRA1** | 3 | GO:0007399, GO:0005488, GO:0005886 |
| **SOX11** | 10 | GO:0007399, GO:0008283, GO:0005488, GO:0031981, GO:0070013, GO:0044428, GO:0043233, GO:0031974, GO:0005737, GO:0005730 |
| **MAPT** | 16 | GO:0007399, GO:0031175, GO:0065008, GO:0048589, GO:0048812, GO:0090066, GO:0048523, GO:0042802, GO:0019904, GO:0019899, GO:0005488, GO:0042995, GO:0043005, GO:0005886, GO:0005829, GO:0005737 |
| **CRIP1** | 3 | GO:0008283, GO:0005488, GO:0005737 |
| **TMSB15A** | 6 | GO:0065008, GO:0007015, GO:0090066, GO:0048523, GO:0005488, GO:0005737 |
| AGR2 | 3 | GO:0065008, GO:0005488, GO:0005737 |
| **ILF3** | 9 | GO:0048523, GO:0005488, GO:0031981, GO:0070013, GO:0044428, GO:0043233, GO:0031974, GO:0005737, GO:0005730 |
| AMD1 | 3 | GO:0016829, GO:0005829, GO:0005737 |
| **CA12** | 4 | GO:0016829, GO:0005488, GO:0005886, GO:0005737 |
| **IQGAP1** | 13 | GO:0019899, GO:0005488, GO:0042995, GO:0031252, GO:0043005, GO:0031981, GO:0005886, GO:0070013, GO:0044428, GO:0043233, GO:0031974, GO:0005737, GO:0005730 |
| MMP1 | 2 | GO:0008237, GO:0005488 |
| NPEPPS | 4 | GO:0008237, GO:0005488, GO:0005829, GO:0005737 |
| CPB1 | 2 | GO:0008237, GO:0005488 |
| **DACH1** | 7 | GO:0005488, GO:0031981, GO:0070013, GO:0044428, GO:0043233, GO:0031974, GO:0005737 |
| **S100A8** | 1 | GO:0005488 |
| SCUBE2 | 1 | GO:0005488 |
| **CACNA1D** | 3 | GO:0005488, GO:0044459, GO:0005886 |
| **TBC1D9** | 1 | GO:0005488 |
| S100A9 | 8 | GO:0005488, GO:0031981, GO:0070013, GO:0044428, GO:0043233, GO:0031974, GO:0005737, GO:0005730 |
| IDUA | 2 | GO:0005488, GO:0005737 |
| MYB | 6 | GO:0005488, GO:0031981, GO:0070013, GO:0044428, GO:0043233, GO:0031974 |
| **NFIB** | 7 | GO:0005488, GO:0031981, GO:0070013, GO:0044428, GO:0043233, GO:0031974, GO:0005730 |
| KDM4B | 1 | GO:0005488 |
| TJP3 | 3 | GO:0005488, GO:0044459, GO:0005886 |
| LASS6 | 2 | GO:0005488, GO:0005737 |
| **DNAJC12** | 1 | GO:0005488 |
| SLC39A6 | 3 | GO:0005488, GO:0005886, GO:0005737 |
| **PROM1** | 3 | GO:0042995, GO:0044459, GO:0005886 |
| DNALI1 | 2 | GO:0042995, GO:0044463 |
| **MTUS1** | 2 | GO:0005886, GO:0005737 |
| **NAT1** | 2 | GO:0005829, GO:0005737 |
| C10orf116 | 0 |  |
| **CYP2B6** | 0 |  |
| BAT2L2 | 0 |  |
